# A Ten-N^6^-Methyladenosine (m^6^A)-Modified Gene Signature Based on a Risk Score System Predicts Patient Prognosis in Rectum Adenocarcinoma

**DOI:** 10.3389/fonc.2020.567931

**Published:** 2021-02-17

**Authors:** Wei Huang, Gen Li, Zihang Wang, Lin Zhou, Xin Yin, Tianshu Yang, Pei Wang, Xu Teng, Yajuan Feng, Hefen Yu

**Affiliations:** ^1^ Beijing Key Laboratory of Cancer Invasion and Metastasis Research, Department of Biochemistry and Molecular Biology, School of Basic Medical Sciences, Capital Medical University, Beijing, China; ^2^ School of Information Science and Technology, University of Science and Technology of China, Hefei, China

**Keywords:** m^6^A, risk score, prognostic prediction, READ, gene signature

## Abstract

**Objectives:**

The study aims to analyze the expression of N^6^-methyladenosine (m^6^A)-modified genes in rectum adenocarcinoma (READ) and identify reliable prognostic biomarkers to predict the prognosis of READ.

**Materials and Methods:**

RNA sequence data of READ and corresponding clinical survival data were obtained from The Cancer Genome Atlas (TCGA) database. N^6^-methyladenosine (m^6^A)-modified genes in READ were downloaded from the “m6Avar” database. Differentially expressed m^6^A-modified genes in READ stratified by different clinicopathological characteristics were identified using the “limma” package in R. Protein-protein interaction (PPI) network and co-expression analysis of differentially expressed genes (DEGs) were performed using “STRING” and Cytoscape, respectively. Principal component analysis (PCA) was done using R. In addition, Gene Ontology (GO) and Kyoto Encyclopedia of Genes and Genomes (KEGG) pathways were used to functionally annotate the differentially expressed genes in different subgroups. Univariate Cox regression analyses were conducted to identify the powerful independent prognostic factors in READ associated with overall survival (OS). A robust likelihood-based survival model was built using the “rbsurv” package to screen for survival-associated signature genes. The Support Vector Machine (SVM) was used to predict the prognosis of READ through the risk score of survival-associated signature genes. Correlation analysis were carried out using GraphPad prism 8.

**Results:**

We screened 974 differentially expressed m^6^A-modified genes among four types of READ samples. Two READ subgroups (group 1 and group 2) were identified by K means clustering according to the expression of DEGs. The two subgroups were significantly different in overall survival and pathological stages. Next, 118 differentially expressed genes between the two subgroups were screened and the expression of 112 genes was found to be related to the prognosis of READ. Next, a panel of 10 survival-associated signature genes including adamtsl1, csmd2, fam13c, fam184a, klhl4, olfml2b, pdzd4, sec14l5, setbp1, tmem132b was constructed. The signature performed very well for prognosis prediction, time-dependent receiver-operating characteristic (ROC) analysis displaying an area under the curve (AUC) of 0.863, 0.8721, and 0.8752 for 3-year survival rate, prognostic status, and pathological stage prediction, respectively. Correlation analysis showed that the expression levels of the 10 m^6^A-modified genes were positively correlated with that of m^6^A demethylase FTO and ALKBH5.

**Conclusion:**

This study identified potential m^6^A-modified genes that may be involved in the pathophysiology of READ and constructed a novel gene expression panel for READ risk stratification and prognosis prediction.

## Introduction

Colorectal cancer (CRC) is one of the most common cancers and a major cause of cancer deaths worldwide. The most recent data reported by Rebecca L. Siegel showed that CRC was the third in terms of cancer incidence and mortality of both men and women in the United States (1). Rectal cancer accounts for about 30% to 35% of all CRC patients, of which most cases have rectum adenocarcinoma (READ) (2). READ is defined as cancer between the dentate line and the junction of the rectosigmoid colon, easy to be diagnosed by digital rectal examination and sigmoidoscopy. Surgery is the standard treatment strategy for early rectal cancer (T1-2 and N0), and neoadjuvant chemoradiotherapy followed by total mesorectal excision (TME) is the treatment for locally advanced (T3-4 and/or N1-2) rectal cancer (3). However, recurrence often occurs after surgery because of its deep pelvic location, complex anatomical relationship, and difficulty in completing the surgery. Mid-lower rectal cancer lies very close to the anal sphincter. It is difficult to preserve the anus and its function during operation. The choice of an appropriate treatment strategy for the rectal cancer depends upon the pathological type, degree of differentiation, depth of tumor infiltration, the presence or absence of regional lymph node (LN) metastasis, and other factors that can predict the invasiveness and prognosis of a tumor (4). Therefore, a deep understanding of the pathological and molecular features of READ is particularly important in predicting prognosis and formulating a clinical treatment plan.

N^6^-methyladenosine (m^6^A) is the most leading posttranscriptional mRNA modification. In mammals, m^6^A is catalyzed by a series of methyltransferases including METTL3/METTL14, WTAP, RBM15/15B, ZC3H13, and KIAA1429—together termed as m^6^A writers (5–10). In this process, a methyl group can be transferred from S-adenosylmethionine (SAM) to an adenosine in mRNA. m^6^A can be removed by demethylases FTO and ALKBH5, which are termed as m^6^A erasers (11, 12). m^6^A methylation can be decoded by multiple m^6^A readers including YTHDF1/2/3, YTHDC1, IGF2BP1/2/3, HNRNPA2B1, HNRNPC, eIF3, FMR1, LRPPRC, and so on (13–19). At the molecular level, m^6^A modifications are dynamically regulated to adjust RNA processes such as alternative splicing, RNA stability, RNA location, and translation (20–24), during both normal cellular processes and under stress or disease conditions (25, 26). Alterations in expressions of m^6^A methylated transcripts subsequently affect cellular function, identity and stemness of the residing cells, determining the cell fate in a context-dependent manner (27–29). Liu et al. reported that m^6^A methylation could induce the activity of Wnt/β-catenin pathway by promoting the expression of CTNNB1 and increasing the proliferation of hepatoblastoma (30). Wu et al. reported that METTL3-mediated m^6^A RNA methylation regulated the fate of bone marrow mesenchymal stem cells and osteoporosis by increasing the translation efficiency of Pth1r (31). However, the role of m^6^A modification in colorectal cancer is still being explored. The expression pattern and the prognostic value of m^6^A-related genes in colorectal cancer has been previously assessed using bioinformatic methods, and the results revealed that the m^6^A-related genes were dysregulated in CRC suggesting that they might play a significant role in the progression of CRC (32, 33). Zhang et al. reported that the m^6^A modification upregulated the expression of CBX8 by maintaining CBX8 mRNA stability and then CBX8 regulated stemness and chemo-sensitivity of colon cancer *via* upregulation of LGR5 (34). The prognostic value of these m^6^A regulators have also been analyzed in CRC, but the prediction accuracy didn’t meet clinical significance (32). Further investigations are required to understand the significance of m^6^A modification in rectum cancer.

Therefore, the aim of this work was to identify the prognostic value of m^6^A methylated transcripts in rectal cancer. The samples of rectal cancer were obtained from The Cancer Genome Atlas (TCGA) database. Differentially expressed m^6^A-modified genes were screened after sample clustering and the prognostic role of these DEGs were further studied. A panel of 10 survival-associated m^6^A-modified genes was constructed. The study may be helpful to understand the clinical significance of this epigenetic modification of RNA in READ and useful for prognostic prediction.

## Materials and Methods

### Database

**Figure 1:**
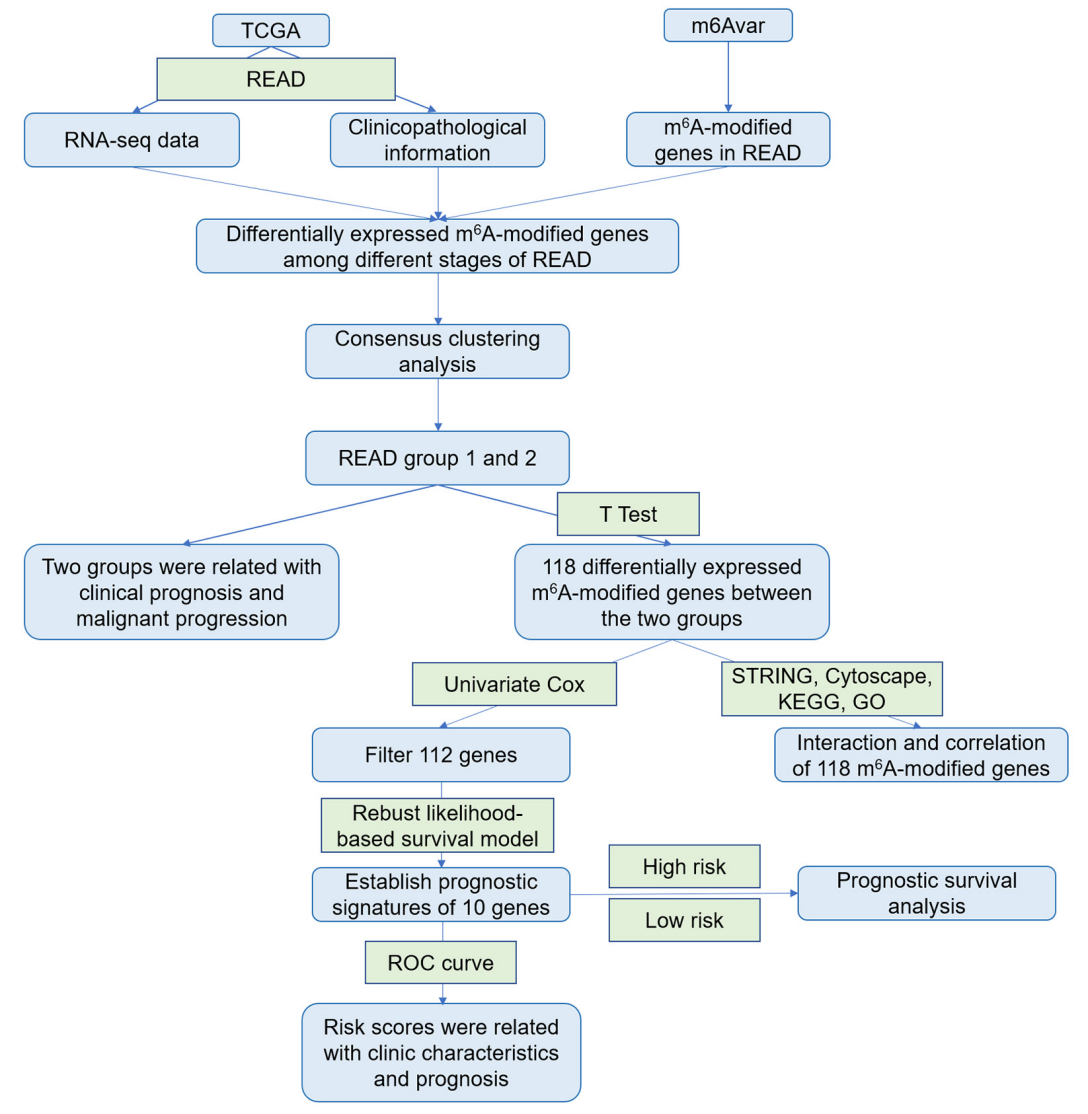
The flowchart of the study.

Rectum adenocarcinoma (READ) RNA-seq transcriptome data (rpkm data) and clinical survival data (including pathological characteristics and survival time) were downloaded from TCGA database (https://cancergenome.nih.gov/), and gene annotation files were downloaded from Genecode (https://www.gencodegenes.org/). Eighty-eight samples of READ with pathological characteristics and RNA-seq expression data were selected. The samples were divided into four categories (Stage I, Stage II, Stage III, Stage IV) according to the pathological characteristics of READ. Clinical information of samples was shown in **Table 1**. m^6^A-modified genes in READ were downloaded from m^6^Avar database (http://m6avar.renlab.org/) (35), and 1,150 m^6^A-modified genes were obtained (**Supplementary Table 1**). RNA-seq data in GSE17536 and GSE27892 were downloaded from the GEO database (https://www.ncbi.nlm.nih.gov/geo/). Our study was designed and analyzed according to the flow chart (**Figure 1**).

**Table 1:** Clinical information of rectal cancer samples from The Cancer Genome Atlas (TCGA) used for analysis.

Clinical factors	Total patients (n = 88)
Stage	
Stage I	13
Stage II	28
Stage III	33
Stage IV	14
Vital status	
Alive	81
Dead	7
Age	
30–40	3
41–50	14
51–60	24
61–70	18
71–80	25
81–90	4

### Data Pre-Processing and Sample Consistency Clustering

Using 88 READ samples with both pathological characteristics and RNA-seq expression data, the expression profiles of 1,047 m^6^A-modified genes (rpkm data) were constructed using gene annotation files. Differentially expressed genes were screened using ANOVA analysis (through “limma” package) among the four types of samples. Benjamini-Hochberg corrected P values < 0.05 and abs(log2 Foldchange) > 1 were selected as thresholds. The DEGs among the READ stages were selected as the m^6^A candidate gene set. The samples were clustered based on the expression of m^6^A candidate gene set by K-means (K=2). Two subgroups were obtained. The age difference between group 1 and group 2 was analyzed by t-test, the WHO grade difference between the two subgroups was analyzed by chi-square test, and Kaplan-Meier overall survival (OS) curves were calculated to compare survival risk between the two subgroups.

### Principal Component Analysis

Principal component analysis (PCA) was performed using “factoextra” package in R to assess the gene expression patterns in the two READ subgroups.

### Screening and Functional Analysis of Differentially Expressed Genes Between Groups 1 and 2

The m^6^A candidate gene expression profiles of group 1 and group 2 were constructed. DEGs were screened using the “limma” package with a cut off criteria set at P < 0.05. Functional annotations in DEGs were done using “clusterProfiler” package in R, which enriched gene ontology and the Kyoto Encyclopedia of Genes and Genomes Pathways. A Protein-protein Interaction network of DEGs was constructed using the Search Tool for the Retrieval of Interacting Genes (STRING, http://string.embl.de/). The combined score higher than 0.70 was regarded statistically significant. Co-expression network of these DEGs was constructed using Cytoscape software.

### Univariate Cox Regression Analysis and Screening Prognostic Signatures

Univariate Cox regression analysis was performed to study the prognostic role of DEGs of m^6^A candidate gene sets in READ. The genes with significant P value were screened as prognostic genes of READ for further analysis.

To screen the characteristic genes most significantly related to the overall survival of rectal cancer, a robust likelihood-based survival model was built using the “rbsurv” package (36, 37). The gene combination with the most frequent occurrence, throughout 1,000 cycles of the robust likelihood based survival model, was assigned as the final prognostic characteristic genes.

### Prediction of Prognosis and Pathological Characteristics Using Risk Score

The risk score for each prognostic characteristic gene in each rectal cancer sample was calculated using the following equation: $\text{Risk}\;\text{Score}\;(\text{patient})=\Sigma_{i=1}^{n}\;Coefi*xi$ where, risk score (patient) is the prognostic characteristic gene risk score for a rectal cancer patient; *Coefi* is the regression coefficient of the prognostic gene that represents the contribution of the gene to the prognostic risk score; *xi* is the expression value of the prognostic gene for each rectal cancer. Based on the risk score, the samples were divided into a high-risk group and a low-risk group. Kaplan-Meier overall survival curves were calculated to compare survival time between the high- and low-risk groups.

### Association of Prognostic Characteristic Genes and Clinical Features

To assess the risk score of prognostic characteristic gene combination that could predict the clinical features of READ, the prognostic genes were mapped to TCGA-READ as combined features. The Support vector machine was used to predict 3-year survival rate, prognosis status and pathological stage of READ using the risk score of combination features. TCGA READ samples were divided into five parts. Five-fold cross-validation was applied. Four-fifths of the samples were used to train the model, which was tested on the test set (the remaining one-fifth of the samples). Then the receiver operating characteristic curve was used to estimate the classification performance. Higher the area under the curve, higher the classification performance.

### Correlation of the Prognostic Characteristic Genes with m^6^A RNA Modification Regulators and Their Expression Patterns in READ Samples

To assess the effect of m^6^A RNA modification regulators on the expression of the 10 prognostic characteristic genes, 13 common m^6^A RNA modification regulators, including METTL3, METTL14, WTAP, FTO, ALKBH5, YTHDC1/2, YTHDF1/2, HNRNPC, KIAA1429, RBM15, ZC3H13, were selected and the expression correlation with the 10 genes were analyzed using Spearman correlation analysis in GraphPad prism 8. The expression correlation among the 10 prognostic characteristic genes was also analyzed using Spearman analysis. We chose the top three genes that were correlated with FTO expression and verified their correlation in GSE17536 and GSE37892 using GraphPad prism 8, the Pearson correlation coefficient and Spearman correlation coefficient were calculated. Moreover, we analyzed the expression patterns of the 10 genes between normal samples and READ samples in TCGA database.

### Statistical Analysis

The t-test was performed to investigate the distribution of risk scores in patients grouped by grade or classification. Univariate Cox regression analyses were applied to identified the prognostic factors and difierent clinicopathological characteristics. Survival curves were plotted by using the “survival” package in R. Long-rank test was used to assess statistical significance. All statistical results with P < 0.05 were regard as statistically significant.

## Results

### Identification of Differentially Expressed m^6^A-Modified Genes in Rectum Adenocarcinoma and Clustering of Patients

**Figure 2:**
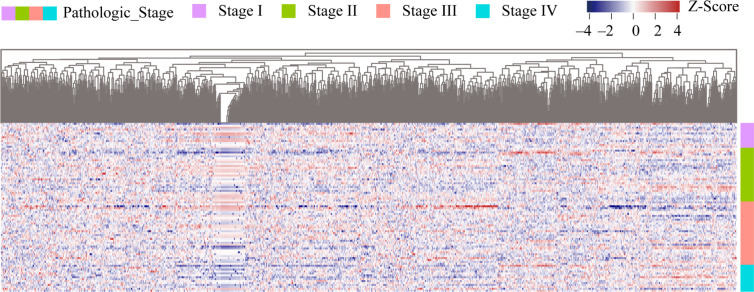
Heatmap of m^6^A candidate gene expression in READ samples among four stages. The samples were sorted by stages from low to high, from I to IV. Stage I samples were represented by the purple group in the right, stage II, III and IV samples by green, red, and blue groups respectively. Genes were sequenced from small to large top-down according to the P value of differential expression analysis results. Red represents high expression of genes, purple represents low expression of genes, and the intensity of the color is directly proportional to the difference of genes.

Using 88 rectum adenocarcinoma samples with both pathological characteristics and RNA-seq expression data, expression profiles of 1,150 m^6^A-modified genes were constructed. Differential analysis was performed to screen differentially expressed genes (DEGs) among four types of samples (Stage I, Stage II, Stage III, Stage IV). With cut-off criteria of P < 0.05 and |log2FC| > 1, a total of 974 differentially expressed genes were screened as m^6^A candidate gene set (**Supplementary Table 2**). A heatmap of differentially expressed m^6^A candidate genes were represented in **Figure 2**. This data revealed that about 90% of m^6^A-modified genes were differentially expressed in READ.

**Figure 3:**
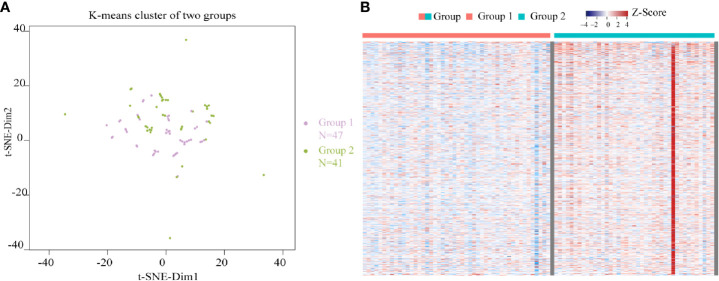
Clustering of READ samples and heatmap of the expression of m^6^A candidate genes between two subgroups. **(A)** K-means clustering graphs of group 1 and group 2. **(B)** The expression heatmap of the 974 m^6^A candidate genes in READ between two subgroups. Row represents a gene while column represents a patient sample. The samples were sorted from left to right with group 1 indicated in red and group 2 in blue. Genes were sequenced from small to large top-down according to the P value from the differential expression analysis results. Red represents high expression, purple represents low expression.

Using the 974 m^6^A candidate genes screened as feature vectors, 88 samples were clustered by utilizing K-means, and two subgroups were obtained: group 1 and group 2. The K-means clustering graph of group 1 and group 2 was shown in **Figure 3A**. As shown in the figure, purple dots represent group 1 and green dots represent group 2. Group 1 was observed to have 47 samples, and group 2 contained 41 samples. The expression heatmap of the 974 m^6^A candidate genes in READ between the two subgroups (**Figure 3B**) indicated that m^6^A candidate genes were highly expressed in group 2.

### Comparative Analysis of Clinicopathological Characteristics and Screening Differentially Expressed Genes Between Two Subgroups

**Figure 4:**
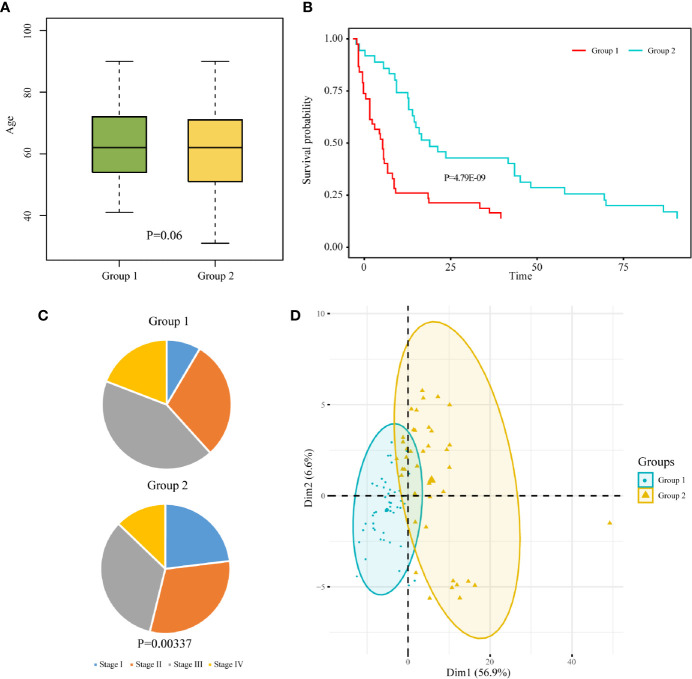
Clinicopathological characteristics between two subgroups of rectal cancer. Box chart of age **(A)**, pathological stage pie chart **(B)**, and survival curve **(C)** between the two subgroups of READ. **(D)** Principal component analysis of the two subgroups.

Samples containing age data were extracted from the two subgroups, and a t-test was performed on the sample age of the two subgroups. The age difference between the two subgroups was not significant (P = 0.06, **Figure 4A**). Samples containing staging data were also extracted from the two subgroups. The difference between the WHO classification of the two subgroups was analyzed by chi-square test. The results showed that there was significant difference between the two subgroups with respect to WHO classification (P = 0.00337). The pie charts of WHO classification between the two subgroups indicated that more than half of the samples in group 1 were in high pathological stages (stage III and stage IV), while more than half of the samples in group 2 were in low pathological stages (stage I and stage II) (**Figure 4B**). Samples containing survival data were extracted from the two subgroups. Kaplan-Meier survival curve showed that the patients in group 2 had significantly better overall survival than those in group 1 (**Figure 4C**). Principal component analysis comparing the expression profiles of the two subgroups showed that there were obvious differences between group 1 and group 2 (**Figure 4D**).

**Figure 5:**
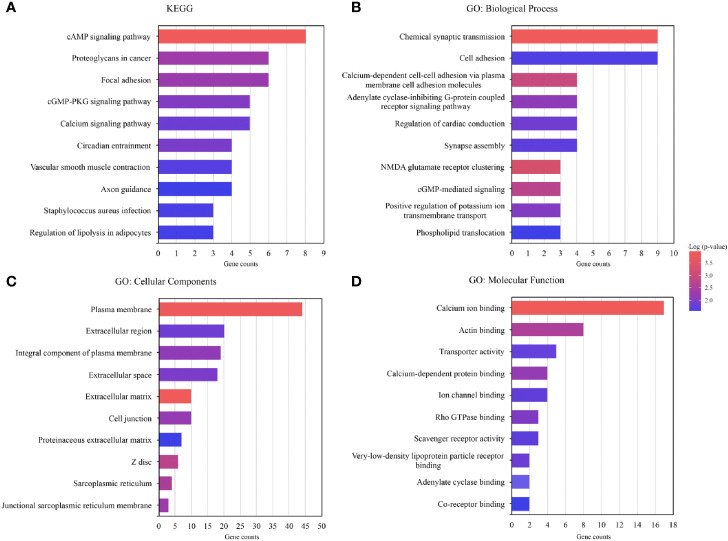
Functional annotation of the genes with differential expression in the two subgroups. **(A)** Functional annotation of the differentially expressed genes (DEGs) using Kyoto Encyclopedia of Genes and Genomes (KEGG) pathway. **(B–D)** Functional annotation of the DEGs using Gene Ontology (GO) terms.

DEGs between the two subgroups were screened using t-test (P<0.05) identifying 118 of them (**Supplementary Table 3**), of which most genes were more highly expressed in group 2 than in group 1. The enrichment analysis of the 118 genes (GO, KEGG pathway) was performed using “clusterProfiler” package in R. 118 DEGs were involved in many signal pathways, especially in cancer development and metastasis, including cAMP signaling pathway, focal adhesion, and so on (**Figure 5A**). The results of the GO analysis were associated with cancer-metastatic processes, including cell adhesion, G-protein coupled receptor signaling pathway, and so on (**Figures 5B–D**). The enrichments indicated that these genes played an important role in the development of cancer.

Co-expression network of these DEGs were visualized *via* Cytoscape. As shown in **Supplementary Figure 1**, most of these genes were co-expressed. The interrelationships between the 118 differentially expressed m^6^A candidate genes were retrieved from STRING database to construct a PPI network. The result illustrated that the hub genes, including NRXN1, ANK2, LPHN2, APOE, TLR8, ESR1, and so on, might be critical for rectum adenocarcinoma, and m^6^A modification could regulate the abnormal expression of these genes (**Supplementary Figure 2**).

### Screening of Survival Associated Genes Using Cox Regression Analysis, Featured Survival Associated Genes, and Functional Annotation

**Figure 6:**
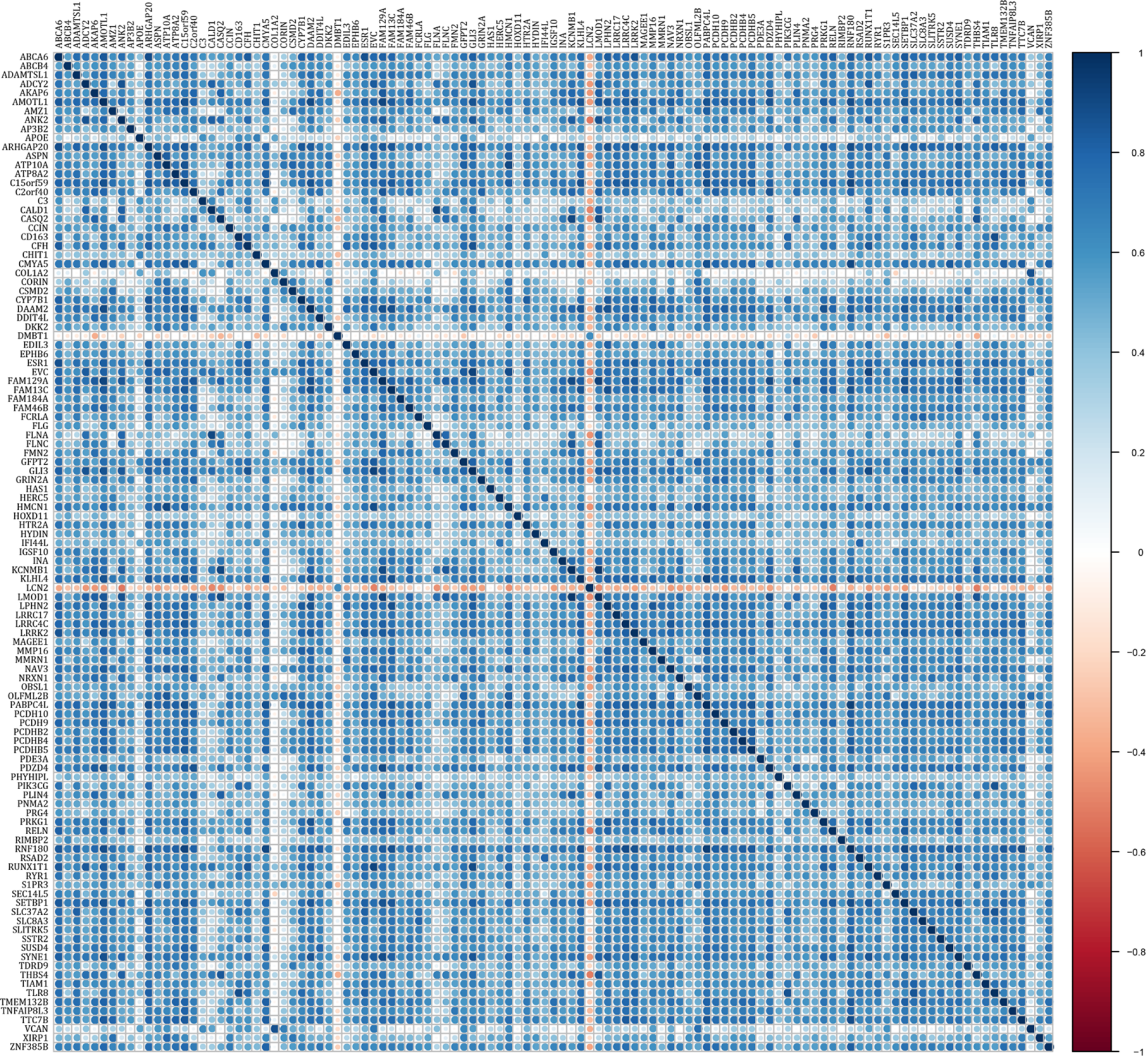
Expression correlation of genes correlated with prognosis of rectal cancer.

To study the prognostic role of m^6^A RNA methylation candidate genes in rectal cancer, the above DEGs were further analyzed by univariate Cox regression using “survival” package in R. The results showed that 112 of the 118 candidate genes were significantly correlated with overall survival in rectal cancer (P < 0.05), see **Supplementary Table 4**. **Table 2** indicated the top 20 genes that significantly influenced the prognosis of rectal cancer. The expression correlations of the 112 prognostic genes were analyzed. Except DMBT1 and LCN2 that negatively correlated with the other genes, most of the genes expressed positively correlated with the others (**Figure 6**).

**Table 2:** Top 20 genes that influence prognosis significantly.

Gene symbol	Description	Cox P value
GFPT2	Glutamine-fructose-6-phosphate transminase 2	6.51E-09
HMCN1	Hemicentin 1	3.26E-08
OLFML2B	Olfactomedin like 2B	8.93E-08
S1PR3	Sphingosine-1-phosphate receptor 3	5.51E-07
NAV3	Neuron navigator 3	6.34E-07
HTR2A	5-hydroxytryptamine receptor 2A	7.45E-07
CYP7B1	Cytochrome P450 family 7 subfamily A member 1	8.38E-07
VCAN	Versican	1.03E-06
ATP10A	ATPase phospholipid transporting 10A	1.14E-06
EVC	EvC ciliary complex subunit 1	1.41E-06
ESR1	Estrogen receptor 1	2.83E-06
C3	Complement C3	3.67E-06
ASPN	Asporin	3.74E-06
PABPC4L	Poly(A) binding protein cytoplasmic 4 like	5.12E-06
CD163	CD163 molecule	8.28E-06
FAM184A	Family with sequence similarity 184 member A	8.66E-06
MMP16	Matrix metallopeptidase 16	1.02E-05
SYNE1	Spectrin repeat containing nuclear envelope protein 1	1.61E-05
CFH	Complement Factor H	1.89E-05
AMZ1	Archaelysin Family Metallopeptidase 1	2.71E-05

There were too many candidate genes that were significantly related to the OS of rectal cancers, which made their usage in clinical diagnosis cumbersome and impractical. Therefore, a robust likelihood-based survival model was constructed to screen the characteristic genes, using “rbsurv” package in R. Through 1,000 cycles of the robust likelihood based survival model, the gene panel with the most frequent occurrence was found as the final prognostic characteristic gene signature, as shown in **Table 3**.

**Table 3:** Survival-associated gene signature screening using forward selection.

Gene symbol	Gene full name	Cox P value
ADAMTSL1	ADAMTS like 1	0.001766
CSMD2	CUB and Sushi multiple domains 2	3.32E-05
FAM13C	Family with sequence similarity 13 member C	0.000341
FAM184A	Family with sequence similarity 184 member A	8.66E-06
KLHL4	Kelch like family member 4	0.000204
OLFML2B	Plfactomedin like 2B	8.93E-08
PDZD4	PDZ domain containing 4	0.000875
SEC14L5	SEC14 like lipid binding 5	0.025102
SETBP1	SET binding protein 1	0.000401
TMEM132B	Transmembrane protein 132B	0.002578

### Characteristics of Prognostic Survival-Associated Gene Signature in READ

**Figure 7:**
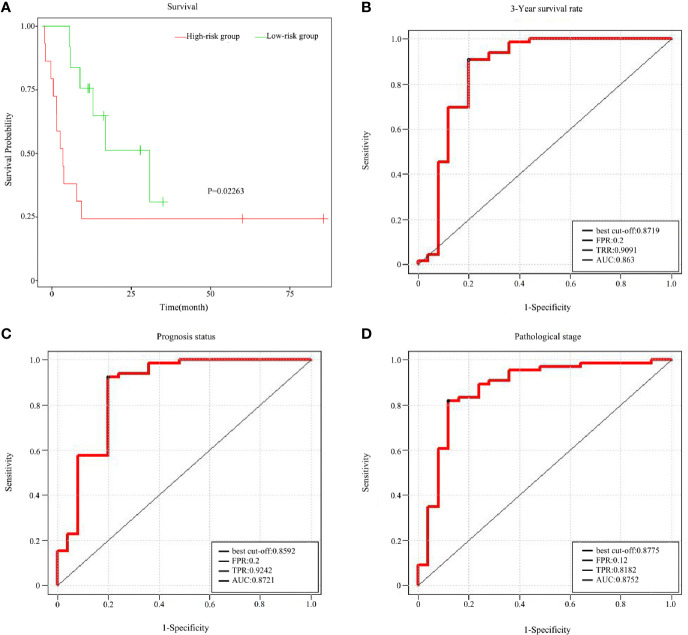
Prognostic value of the survival-associated gene signature in READ. **(A)** Kaplan–Meier overall survival curves for READ patients classified into high- and low- risk groups based on the risk score calculated from 10 survival-associated genes. Receiver operating characteristic (ROC) curves displayed the predictive power of the risk Score for the 3-year survival rate **(B)**, prognosis status **(C)**, and pathological stage **(D)**.

To assess the prognostic value of the ten featured survival-associated genes, a survival-associated gene signature was constructed by integrating the expression of these featured survival-associated genes using a regression coefficient. Then, a risk score for each patient was calculated and the patients were ranked based on increasing score, after which patients were classified into a high-risk or a low-risk group based on the median risk score. Kaplan-Meier survival analysis showed that there was a significant difference in the OS between the high-risk group and the low-risk group (P = 0.02263, **Figure 7A**), supporting the value of risk score in identifying the prognostic risk among the samples.

To further assess the predictive accuracy of the ten featured survival associated genes using the risk score of a combination of features, these ten genes were mapped to the TCGA-READ as combination features. Support vector machine was used to predict 3-year survival rate, prognosis status and pathological stage of READ by the risk score of combination features. The predictive accuracy for 3-year survival rate was remarkable, yielding an AUC value of 0.8630 in a validation cohort (**Figure 7B**). The prediction effect on prognosis status was shown in **Figure 7C**. The area under the AUC line for the validation set was 0.8721, which was also quite remarkable. Likewise, the predictive accuracy for pathological stage was quite good, because the AUC value was 0.8752 (**Figure 7D**). These results were quite encouraging and highlight the potential clinical significance of the ten survival associated gene signature for the prediction of the poor outcomes in READ patients.

### Correlation Between the Survival-Associated Genes and the m^6^A RNA Modification Regulators and the Expression Patterns of the Survival-Associated Genes in READ

**Figure 8:**
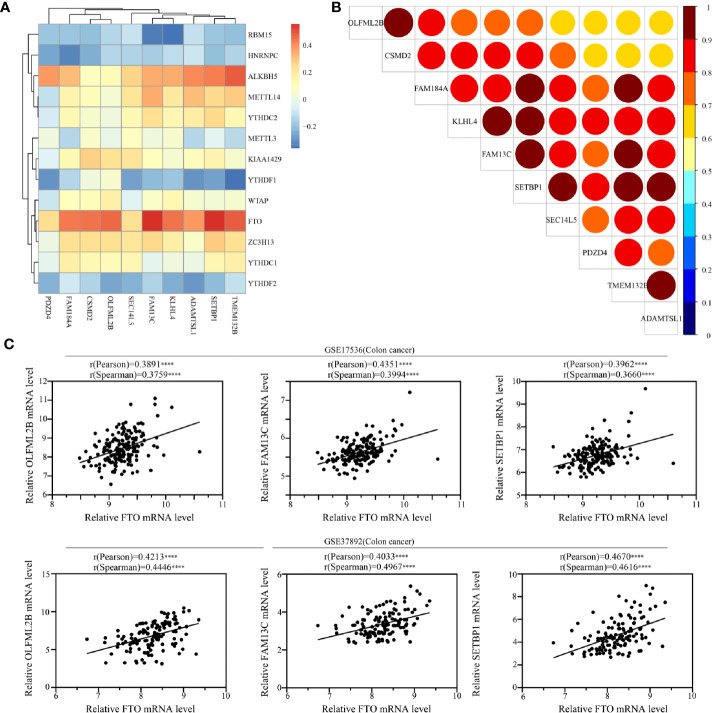
Correlation between the survival-associated genes and the m^6^A RNA modification regulators. **(A)** Correlations between m^6^A regulators and the survival-associated genes in READ cohort using Spearman analysis. **(B)** Correlations between the survival-associated genes in READ cohort using Spearman analysis. **(C)** Correlations between FTO and the survival-associated genes OLFML2B, FAM13C, and SETBP1 in Gene Expression Omnibus (GEO) data sets using Spearman analysis (****P < 0.0001).

The ten featured survival-associated genes were selected from m^6^A modified genes. To better understand the characteristics of this gene signature, we analyzed the correlation between the ten genes and m^6^A RNA modification regulators using spearman correlation analysis. The data showed that m^6^A erasers FTO and ALKBH5 were positively correlated with most genes (**Supplementary Figure 3**), while m^6^A readers RBM15, HNRNPC, and YTHDF2 had negative correlation with certain genes to some degree (**Figure 8A**). The detailed correlation coefficients were shown in **Supplementary Table 5**. In addition, we also tested the correlation among each of the 10 genes. The data showed that the ten genes were highly correlated to each other (**Figure 8B**). Moreover, from the ten genes we chose the top three genes that were correlated with FTO expression and further verified their correlation in GEO data sets (GSE17536 and GSE37892). The results revealed a significantly positive correlation between the expression of three featured survival-associated genes and the expression of FTO (**Figure 8C**). Taken together, these results demonstrated that these ten genes may be coordinately regulated in READ involving m^6^A modification.

Moreover, we analyzed the expression patterns of the 10 genes in normal samples and READ samples in TCGA database. The results showed that most of the genes, except CSMD2 and OLFML2B, had lower expression in READ samples than in normal samples (**Supplementary Figure 4**). The expression levels of OLFML2B had no significant difference between normal samples and cancer samples, while the expression levels of CSMD2 were higher in cancer samples than in normal samples.

## Discussion

Rectal cancer is one of the most common cancers. The cure rate and the overall survival rate after resection are poor, and the local recurrence rate is high. In recent years, finding the prognosis-related gene markers and developing other prediction methods of rectal cancer have become popular research hotspots (38–41). For example, by comparing the immune cell composition of 870 colon cancer patients and 70 normal people, Zhou R et al. constructed a diagnostic model, diagnostic immune risk score (dirs), and a prognostic immune risk score (PIRS), which is an independent prognostic factor of relapse free survival rate of each series, showing a better prognostic value than TNM stage (39). In 1965, Dr. Joseph Gold found a protein, called carcinoembryonic antigen (CEA), in the blood of colon cancer patients, which usually exists in the gastrointestinal tissue during the development of fetus (42). This protein is now used as a biomarker for the diagnosis and prognosis of colorectal cancer (43). However, most biomarkers show limited sensitivity, none with AUC value above 0.85. Therefore, a more reliable prediction method for the prognosis of different patients is needed, so as to bring about more effective treatments for patients.

Therefore, toward this, in this study we constructed a survival-associated gene signature to predict the prognosis of READ using m^6^A-modified genes as the candidate set. This gene signature was good at distinguishing the prognostic risk among the samples. The predicative accuracy for prognosis of READ was remarkable, especially with respect to the 3-year survival rate (AUC = 0.8630) and prognostic status (AUC=0.8721).

A growing number of evidences have shown that m^6^A RNA modifications are related to tumorigenesis, invasion and metastasis (10, 25, 44). The m^6^A modification regulators can act either as carcinogenic or anticancer genes in malignant tumors (10). In fact, their precise roles and mechanisms are widely studied. The prognostic value of these regulators have also been analyzed in multiple malignant tumors, including liver cancer (45), gastric cancer (46), clear cell renal cell carcinoma (ccRCC) (47, 48), colorectal cancer (32). The prognostic signature obtained using two m^6^A RNA methylation regulators (METTL14 and METTL3) had significant value in ccRCC, the AUC value for their risk prediction is 0.706 (47, 48). The expression of METTL14 and ALKBH5 were reported to be independent prognostic factors in CRC (32). Liu T et al. reported that YTHDF1 and HNRNPC can be used as prognostic factors of colon cancer and the AUC value for prognosis was 0.62 (33). However, there are some limitations in these previous studies: For example, most of the above-mentioned studies of cancer prognosis are based on the differential expression of m^6^A regulators. Although they have achieved satisfactory results, the diversity of m^6^A regulators is quite different from that of m^6^A modified genes. Therefore, the specificity of the prediction is poor, and the prediction accuracy will be low.

In the present study, m^6^A-modified genes were selected as the candidate genes to construct the prognosis predictive model. Firstly, expression profiles of 1,047 m^6^A-modified genes were constructed and 974 differentially expressed genes among four types of READ samples were screened. Then, two READ subgroups (group 1 and group 2) were identified by consensus clustering based on the expressions of DEGs. The two subgroups were significantly different in overall survival and pathological stages. Next, we screened 118 DEGs between the two subgroups and found that the expression profiles of 112 genes were related to prognosis. Next, a survival-associated signature of ten genes (ADAMTSl1, CSMD2, FAM13C, FAM184A, KLHL4, OLFML2B, PDZD4, SEC14L5, SETBP1, TMEM132B) was retrieved from the 112 genes. The signature performed very well for prognosis prediction of READ. The predictive accuracy of this gene panel is better than that of other researches, yielding an AUC value of above 0.85.

ADAMTSL1, a secreted glycoprotein and a member of the ADAMTS (a disintegrin and metalloproteinase with thrombospondin motif) family, is a component of the extracellular matrix (49). ADAMTSL1 is primarily expressed in skeletal muscle, and is also expressed in other tissues. It is hypermethylated in ER-positive breast cancer (50). But its function in cancers is unclear. ADAMTSL1 was reported to be associated with prognosis of breast cancers in young women (51). CSMD2 (CUB and sushi multiple domain protein 2) was thought to be involved in the control of complement cascade of the immune system. Defects in this gene have been associated with schizophrenia (52). CSMD2 was reported to be a tumor suppressor, its low expression was significantly associated with differentiation, lymphatic invasion, tumor size, and the poor prognosis of colorectal cancer patients (53). FAM13C (Family with sequence similarity 13, Member C), one of the FAM protein family, may be involved in intracellular signal transduction pathways relevant for cancer based on sequence analyses (54). FAM13C overexpression was an independent prognostic marker in prostate cancer (55). The biological function of FAM184A is unknown, but it was found to be increased in endometrial cancer and was classified as a risky prognostic gene (56). KLHL4 is a member of the KLHL protein family and associates with a disorder known as X-linked cleft palate (CPX). KLHL4, as a novel p53 target gene, was reported to inhibit cell proliferation by activating p21^WAF/CDKN1A^ (57). Its high expression in glioblastoma contributed to prognosis analysis (58). OLFML2B (Olfactomedin-Like 2B) is an olfactomedin domain-containing protein and may act as an oncogene in the development of gastric cancer. The overexpression of OLFML2B in gastric cancer may be used as a novel diagnostic and prognostic target for GC (59). PDZK4, PDZ domain containing protein 4, was upregulated in synovial sarcomas and induced proliferation of synovial sarcoma cells (60). PDZK4 had a lower level in liver cancer tissues than adjacent normal tissues (61), which was consistent with our result. SEC14L5, belonging to the subgroup of SEC14-containing proteins, was found to be significantly altered in the non-child abused PTSD patients (62). Its roles in cancers have not been reported yet. SETBP1 (SET Binding Protein 1), which encodes an AT-hook transcription factor, played a significant role in driving human primary AML development. Its overexpression was associated with poor prognosis of human AMLs (63). While SETBP1 can reduce the progression of NSCLC as a tumor suppressor and can be used for a prognostic biomarker in NSCLC (64). TMEM132 is a transmembrane protein, while the molecular functions of TMEM132 remain poorly understood and underinvestigated. It may be an ancient architecture of cohesin and immunoglobulin domains, belong to neural adhesion molecules (65).

The method that we have provided in this study is not only helpful to the formulation of postoperative treatment plan for rectal cancer patients, but also can be used to screen the high-risk population of rectal cancer, so as to achieve the purpose of early detection and treatment, and improve the survival period. Although the gene spectrum proposed in this paper has achieved an ideal result in predicting the prognosis of rectal cancer, there are still some limitations in this study. The main problem is the number of samples as we obtained only 88 samples from TCGA database. In order to improve the accuracy of the prediction model, more samples are required in the next study to further improve the gene spectrum, in addition to external data from other databases to strengthen the validity of the model. Furthermore, the present study is purely computational and so future experimental and clinical data are needed to validate our results.

In conclusion, our study revealed that a panel of ten survival-associated genes could be used for predicting the prognosis of rectal cancer. We have also provided a new strategy to screen the prognostic factors from m^6^A-modified genes.

## Data Availability Statement

Publicly available datasets were analyzed in this study. These data are available from The Cancer Genome Atlas (TCGA) database (https://cancergenome.nih.gov/), RMVar (http://rmvar.renlab.org), and GEO datasets (https://www.ncbi.nlm.nih.gov/geoprofiles/).

## Author Contributions

WH, YF, and HY contributed conception and design of the study. XY, TY, and PW organized the database. GL, ZW, and LZ performed the analysis and drew the figures. WH wrote the first draft of the manuscript. XT wrote sections of the manuscript. HY contributed to manuscript revision and proofreading. All authors contributed to the article and approved the submitted version.

## Funding

This study was supported by the National Natural Science Foundation of the People’s Republic of China (grant no. 81672726, no. 41931291, no. 81902960) and the Nature Science Foundation of Beijing (grant no. 7204241).

## Conflict of Interest

The authors declare that the research was conducted in the absence of any commercial or financial relationships that could be construed as a potential conflict of interest.
