# Supplementary material for: A Ten-N6-Methyladenosine (m6A)-Modified Gene Signature Based on a Risk Score System Predicts Patient Prognosis in Rectum Adenocarcinoma
Source: Front Oncol. 2021 Feb 17;10:567931. doi: 10.3389/fonc.2020.567931 (PMC7925823; doi:10.3389/fonc.2020.567931)
Supplement: Supplementary file 1 [file DataSheet_1.pdf]

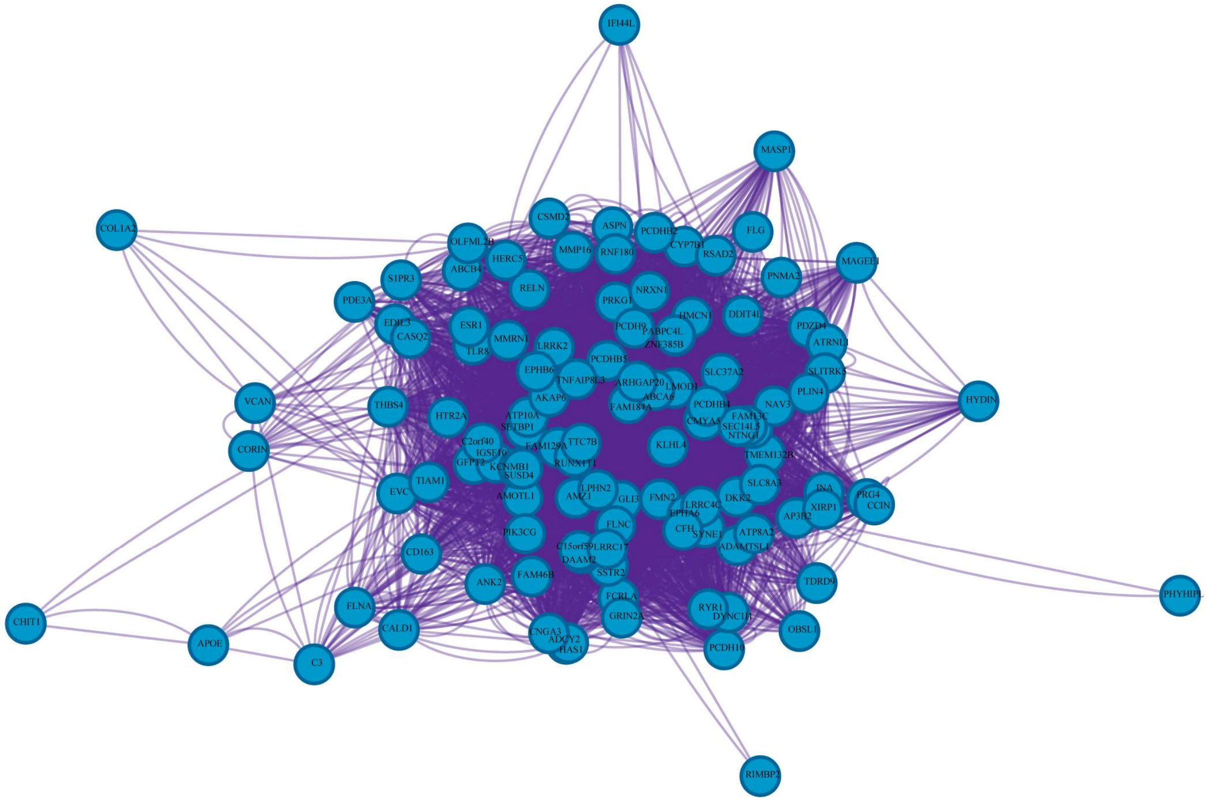

Supplementary Figure 1. Co-expression network of the differentially expressed genes visualized via Cytoscape software.

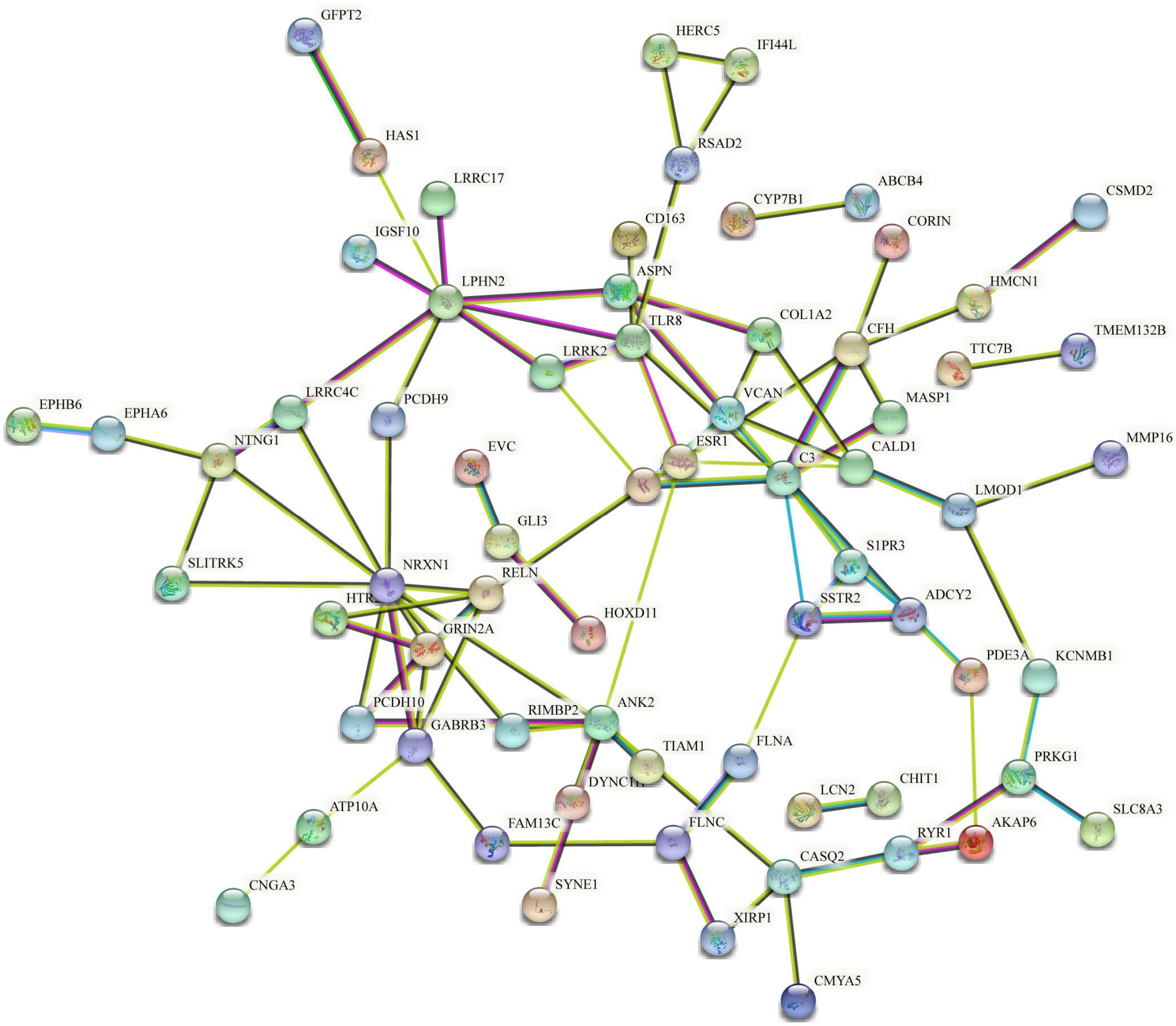

Supplementary Figure 2. The Protein-protein Interaction (PPI) network of 118 differentially expressed m^{6}A candidate genes constructed using the "STRING" database.

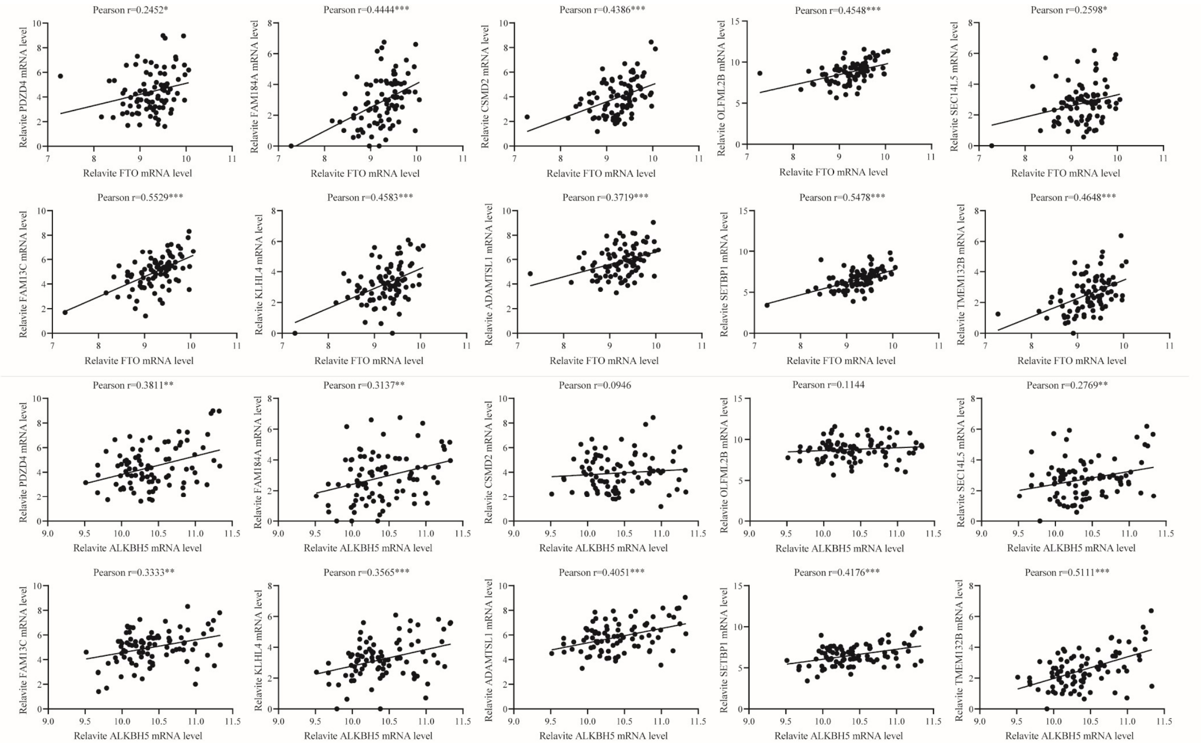

Supplementary Figure 3. The correlation of FTO and ALKBH5 with the 10 survival-associated genes. The expression data of the 10 featured survival-associated genes and the expression data of FTO and ALKBH5 in READ samples from TCGA were used to analyse their correlation using spearman correlation analysis (*P<0.05, **P<0.01, ***P<0.001).

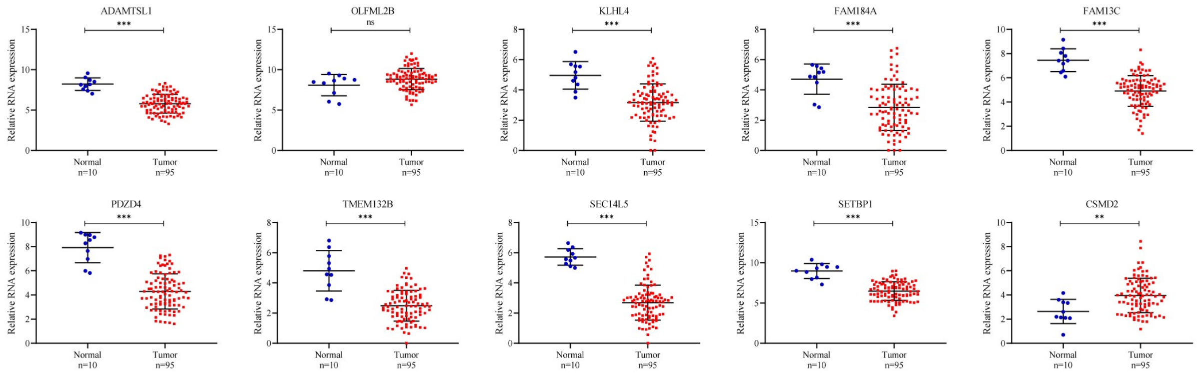

Supplementary Figure 4. Expression patterns of the survival-associated genes in READ samples in TCGA database (*P<0.05, **P<0.01, ***P<0.001).
